# Me, Myself, and I: Neural Activity for Self versus Other across Development

**DOI:** 10.3390/children10121914

**Published:** 2023-12-12

**Authors:** Paola Zanchi, Jean-Baptiste Ledoux, Eleonora Fornari, Solange Denervaud

**Affiliations:** 1 Department of Diagnostic and Interventional Radiology, Lausanne University Hospital, University of Lausanne, 1005 Lausanne, Switzerland; 2 CIBM Center for Biomedical Imaging, 1015 Lausanne, Switzerland; 3 MRI Animal Imaging and Technology, Polytechnical School of Lausanne, Swiss Federal Institute of Technology Lausanne (EPFL), 1015 Lausanne, Switzerland

**Keywords:** self-vs.-other perception, social cognition, development, neural activity, social processing

## Abstract

Although adults and children differ in self-vs.-other perception, a developmental perspective on this discriminative ability at the brain level is missing. This study examined neural activation for self-vs.-other in a sample of 39 participants spanning four different age groups, from 4-year-olds to adults. Self-related stimuli elicited higher neural activity within two brain regions related to self-referential thinking, empathy, and social cognition processes. Second, stimuli related to ‘others’ (i.e., unknown peer) elicited activation within nine additional brain regions. These regions are associated with multisensory processing, somatosensory skills, language, complex visual stimuli, self-awareness, empathy, theory of mind, and social recognition. Overall, activation maps were gradually increasing with age. However, patterns of activity were non-linear within the medial cingulate cortex for ‘self’ stimuli and within the left middle temporal gyrus for ‘other’ stimuli in 7–10-year-old participants. In both cases, there were no self-vs.-other differences. It suggests a critical period where the perception of self and others are similarly processed. Furthermore, 11–19-year-old participants showed no differences between others and self within the left inferior orbital gyrus, suggesting less distinction between self and others in social learning. Understanding the neural bases of self-vs.-other discrimination during development can offer valuable insights into how social contexts can influence learning processes during development, such as when to introduce peer-to-peer teaching or group learning.

## 1. Introduction

“Is that me or you?”. From playgrounds to boardrooms, the ability to form an accurate self-vs.-other representation enables individuals to navigate social interactions by understanding their thoughts, feelings, and intentions as distinct from others [1].

This discriminative skill affects the capacity to embrace others’ perspectives and feelings in a non-centered manner. We use this information to form and maintain constructive social interactions (i.e., friendships, romantic partnerships, and professional networks) [2,3] and to navigate complex social environments using self-vs.-other cues adequately [4]. Impaired discriminative skills across development impact social cognition, from recognizing oneself in the mirror to interpreting the intentions and emotions of others [5,6].

Given the importance of well-developed self-vs.-other discriminative skills, unveiling underlying brain activation maps across development could help to address in an informed manner the needs and capacities at different stages of life. While different studies shed light on either behavioral or neuroimaging makers of this discriminative ability at different ages or by contrasting adults with young children, none report a cross-sectional perspective on the neural activity across development.

The development of self-vs.-other representation is a complex process influenced by different factors, including genetics, brain maturation, and environmental experiences [7,8]. During the first years of life, the ability to distinguish the self from the environment and from others starts emerging, mostly regarding the physical self [9,10]. Babies develop their sensorimotor ability while experiencing different perceptual and sensorimotor events, such as self-produced sounds or touching their face and body. These events contribute to uniquely specifying and separating their own body as differentiated entities from the environment [11,12].

Then, discriminative abilities become more abstract. Infants as young as 20–24 months old have been shown to display self-recognition in mirror tasks (i.e., touch their forehead and not their reflection). More interestingly, this ability comes along with the ability to discriminate their reflection as distinct from other objects or people [13,14]. This initial phase lays the foundation for more complex self-vs.-other differentiations that will develop in later years.

As children progress through early childhood, their brain elaborates increasing preciseness and stability in behavior [15,16], while sophisticating their self-concept, incorporating beliefs and attitudes about their abilities, personality traits, and social identities [17,18]. At the same time, their understanding that others can have beliefs and perspectives different from their own (Theory of Mind (ToM)) begins to develop, which contributes to the development of empathy and social cognition [19]. During this phase, the cognitive processes related to self-concept and identity continue to evolve, and the teenage years mark a period of increased self-awareness and the exploration of one’s identity within a group [20].

Adolescence is particularly important for the development of self-vs.-others’ representations, with significant changes in social and emotional abilities, reflected in new relationships and social roles. Social comparisons become more important, shaping individuals’ self-esteem, and influencing the way individuals perceive themselves compared to others [21]. Adolescents’ brains undergo significant changes during this period, notably in regions involved in social cognition and emotion regulation (i.e., the posterior superior temporal sulcus and the medial prefrontal cortex) [22].

Throughout adulthood, the development of self-vs.-other representation continues to evolve, and the cognitive and emotional processes may change as people age, affecting their ability to understand and adapt to social interactions [3]. The matured self-vs.-other representation becomes crucial in guiding social interactions, decision-making, metacognition, and emotional regulation [1,23,24]. The neural substrates associated with self-referential thinking and social cognition demonstrate a more stable and integrated pattern of activation [25].

These transformations of self and other representations across development are reflected in the activity of specific brain areas across various stages of development. For example, distinct patterns of brain activation have been observed between adults and children in the developmental trajectory of ToM reasoning. In adults, brain regions such as the temporoparietal junction (TPJ), the superior temporal sulcus (STS), the precuneus (PC), the temporal poles, the medial prefrontal cortex (mPFC), and the posterior cingulate have been associated with the explicit ToM [26,27,28,29].

Over childhood, it has been demonstrated that the right TPJ shows a developmental shift and becomes more selective. While it initially responds equally to both mental and nonmental social information, as children age it exhibits a more robust response to mental-state information [28]. In addition, changes in activation within the mPFC (i.e., ToM-related regions) have been observed in children aged between 9 and 16 years old, with a shift from more ventral to more dorsal regions of the mPFC [30]. As the ventral mPFC is associated with self-referential thought and the dorsal mPFC with cognitive control, this developmental shift might indicate a move from ToM reasoning based on simulation to a more detached and top-down approach [31].

While there is a gradual gain in social cognitive processing, a deeper understanding of self-vs.-other discrimination across development could shed light on differences in social needs and improve the social dimension of learning environments (e.g., when is peer learning a good strategy?). Indeed, the quality of early social experiences is critical and predictive of the development of self and other representations [32,33]. Providing adequate social feedback may help shape emotion recognition and regulation as they develop social skills such as empathy and perspective-taking [33]. This knowledge should have diverse implications, influencing various educational strategies, guiding therapeutic interventions, and enriching our comprehension of social dynamics across the entire lifespan.

This study aimed to explore age-related brain activity differences in the processing of self-vs.-other stimuli. A total of 39 participants from 4-year-olds to adults were separated into four different groups according to their age. Participants underwent an fMRI task session to evaluate their brain activity in response to self-vs.-other stimuli. Three main hypotheses were explored: (1) brain activity related to self-perception: we hypothesized activity for self-vs.-other stimuli in areas associated with adults’ self-recognition, including the frontal, parietal, and occipital regions [25], as well as the ACC and the posterior cingulate cortex (PCC) [34,35,36,37,38]; (2) brain activity related to ‘other’ perception: We expected increased brain activity elicited within the precuneus and the angular gyrus (temporoparietal junction (TPJ), the superior temporal sulcus (STS), the temporal poles, and the medial prefrontal cortex (mPFC)) as these areas tend to be more active when looking at others compared to oneself [39,40]. Furthermore, we predicted higher activity for stimuli related to others compared to self in the temporal cortex and regions associated with recognizing visual stimuli. These areas have been previously linked to the early identification of humans and human movement [41]; finally, (3) developmental changes: We hypothesized to observe an overall linear increase in activity across development given the gradual gain in self-vs.-other discriminative skills [3]. However, we also expected non-linear changes to happen in middle age children (7–10 y.o.) for self stimuli as well as in teenagers for other stimuli (11–19 y.o.) given the non-linear developmental trajectory across middle age children in social cognition [42], as well as the known changes in self-related information over the teenage years [20].

## 2. Method

### 2.1. Participants

Based on past work implying a task-based fMRI acquisition in 4–10-year-old children [43], a total of 42 healthy participants were recruited for the experiment. However, three participants were excluded due to poor MR image quality given to excessive movement, leaving a sample of 39 participants for the final analyses (mean age = 13.54, SD = 8.00). To enroll for this study, the participants had to be in the targeted age range: (a) from 4 to 6 years old, (b) from 7 to 10 years old, (c) from 11 to 19 years old, and (d) from 20 to 35 years old (Table 1). Exclusion criteria were neural or behavioral disabilities as checked through a self-reported questionnaire or, for participants <14 years old, through a parental questionnaire. The study was approved by the ethics committee (CER-VD #2018-00244) and written and oral informed consent for the course of the experiment was obtained from the participants, as well as from their parents (when <14 y.o.).

### 2.2. fMRI Video Task

**Figure 1:**
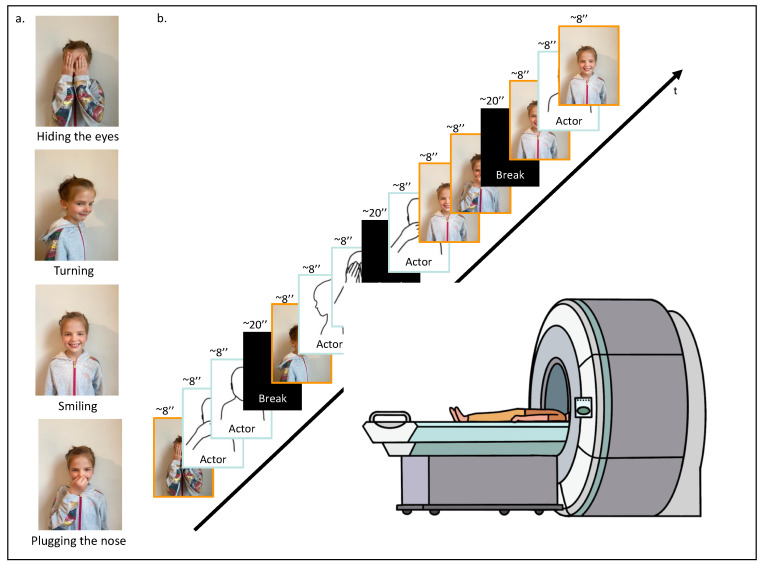
fMRI task design. (**a**) First, the participant was asked to execute four different actions in front of a camera: (1) hiding their eyes with their hands, (2) turning on themselves, (3) smiling, and (4) plugging their nose. (**b**) Second, while being placed in the scanner, the participant was asked to watch the video-based fMRI task; 12 video clips randomly arranged displaying the four different movements performed by the participant or a same-age and same-gender unknown actor.

Each participant was invited to the lab. When arriving, and after having signed the ethics form and completed the questionnaires, the participant was asked to perform four specific actions in front of a camera: (1) hiding their eyes with their hands, (2) turning on themselves, (3) smiling, and (4) plugging their nose (Figure 1a). These actions were recorded for approximately 8 s each. Subsequently, these personalized recordings were incorporated into the fMRI video task, where they were randomly mixed with similar video clips of actions performed by an unknown same-age-same-gender actor. The final personalized fMRI video task was composed of 12 video clips. To ensure between-participants variability and within-participant unpredictability, video clip presentations were randomized, ensuring that each action was shown at least once, and four of them were randomly repeated. A randomization function (numpy.random) was used to ensure that the experimental conditions were presented in a balanced and unbiased manner. The 12 video clips, each lasting approximately 8 s, were divided into four segments, with 20 s breaks in between to offer participants brief rest intervals for basal neural activity recording. In total, the video task lasted 160 s (Figure 1b). The fMRI video task was coded and presented using PsychoPy 2.0 software (October 2020) [44].

### 2.3. Procedure

Participants were placed in the MRI scanner and asked to simply watch the fMRI video task. As described above, the task comprised 12 video clips randomly arranged, featuring four distinct actions performed each by ‘self’ and ‘other’. During the scanning session, the participants were instructed not to move and just to relax and watch the personalized fMRI video. This protocol aimed to capture neural responses associated with passive observation.

### 2.4. MRI Data Acquisition

Anatomical and functional images were collected using a Siemens (Siemens Healthineers, Erlangen, Germany, VE11E Software version) 3T Prisma-Fit MR scanner with a 64-channel head coil in the CIBM Center for Biomedical Imaging of the Lausanne University Hospital (CHUV). MRI sessions were conducted with two different acquisitions for each subject: (i) 3-dimensional T1 weighted MP-RAGE (Magnetization Prepared—RApid Gradient Echo) (TR = 2000 ms, TE = 2.47 ms, 208 slices; voxel size = 1 × 1 × 1 mm^3^, flip angle = 8°), used as an anatomical reference for brain extraction and surface reconstruction, and (ii) a functional MRI (fMRI) acquired continuously using a standard echo-planar gradient echo sequence, with simultaneous multislice (SMS) imaging technique. This technique covers the whole brain with an isotropic voxel size of 2 mm ([TR] = 1000 ms; echo time [TE] = 30 ms; 64 axial slices; slice thickness = 2 mm, no gap between slices, flip angle = 80°, matrix size = 100 × 100, a field of view [FOV] = 200 mm, SMS factor = 4, parallel imaging acceleration factor = 2). The total fMRI acquisition time was 3 min and 12 s (for more details see Table S1).

To prevent head movement and noise, foam pads were placed around the participants’ heads inside the coil.

### 2.5. MRI Data Processing

MRI data were preprocessed using SPM12 software (Wellcome Department of Cognitive Neurology, London, UK) and run on Matlab (Mathworks, Natick, MA, USA, Version 7.13). To address motion artifacts in the functional images, a 6-parameter rigid-body realignment was performed, with the first scan serving as a reference. The realigned images were then slice-timing corrected, and both the functional images and the high-resolution T1-weighted (T1w) anatomical image of the participant were co-registered, using mutual information. Using the anatomical images as an estimation basis, functional images were normalized to the MNI template and spatially smoothed using an 8 mm Gaussian filter.

Data visualization and figure preparation were performed using the xjView Toolbox for SPM (http://www.Alivelearn.net/xjview, accessed on 1 November 2022), designed to explore and present SPM results. An aal atlas was used to label and describe anatomical locations [45].

### 2.6. Neural Activation Analyses

In statistical single-subject analysis, performed by applying a general linear model, neural activities while watching self-vs.-other stimuli were retrieved; onsets were video clips’ starting times, and durations were the 7 s of the video clips. The realignment parameters were included in the model as a nuisance variable. A frequency threshold of 121 Hz was used as a high-pass filter cutoff to filter the amplitude of signals, allowing the removal of the low-frequency noise or interference. A visual inspection of estimated motion parameters was conducted for each participant.

Several recording images of 4 participants were excluded according to the artifact repair procedure performed using ArtRepair [46], an SPM toolbox for motion adjustment, data repair, and noise filtering. The exclusion criteria were based on participant-based significant movement artifacts identified during the inspection of motion parameters. Statistical inference from the acquired group was performed using the first-level contrasts of interest as input values. To test hypotheses (1), brain activity related to self-perception, and (2), brain activity related to ‘other’ perception, a mixed model ANOVA was computed with, as intra-subject variables, the contrasts representing brain activity during perception of self and others (two levels) at the whole-brain level across age groups as inter-subject variables (four levels). A threshold of *p* < 0.05 (FDR corrected) was used for the selection of the significant clusters. Hypothesis (3) regards developmental changes. To investigate and reveal the effect of self-vs.-other perception between the different age groups, we performed a post hoc comparison using paired *t*-tests on BOLD signal changes extracted from significant clusters. The paired *t*-tests were conducted to compare the activation of the brain region during self-vs.-other perception, for each age group. Given that we tested within participant values, based on their FDR-corrected data, paired *t*-tests were uncorrected.

## 3. Results

### Neural Activation Analyses

This study aimed to explore age-related differences in brain activity during the processing of self-vs.-other stimuli. Generally, we expected (1) increased brain activity associated with self-perception across development in regions implied in self-recognition in adults; (2) increased brain activity related to ‘other’ stimuli in areas associated with social cognition and regions associated with the recognition of visual stimuli; and (3) an overall linear increase in activity across development, as well as a non-linear changes in middle age children for self-perception and in teenagers for ‘other’ stimuli.

**Figure 2:**
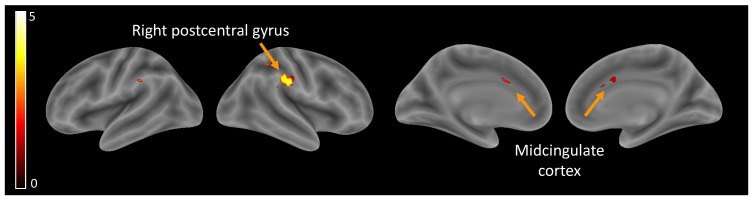
Brain activity related to self-perception.

Hypothesis (1), brain activity related to self-perception: Independently of the age group, self-perception compared to ‘other’ perceptions elicited higher activity in the midcingulate cortex (MCC) and the right postcentral gyrus (Table 2, Figure 2).

**Figure 3:**
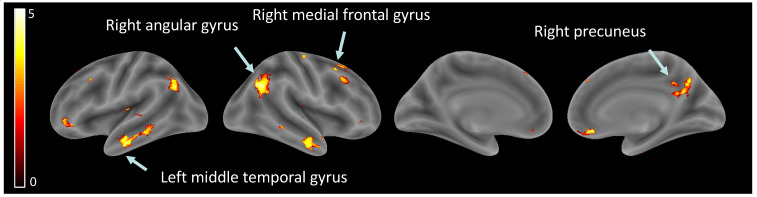
Brain activity related to ‘other’ perception.

Hypothesis (2), brain activity related to ‘other’ perception: Significant increases were observed in several brain regions when observing other than self, within the right angular gyrus, left angular gyrus, right inferior temporal gyrus, left middle temporal gyrus, right rectus, right precuneus, right medial prefrontal cortex, right superior frontal gyrus, and left inferior orbital gyrus (see Table 3, Figure 3).

**Figure 4:**
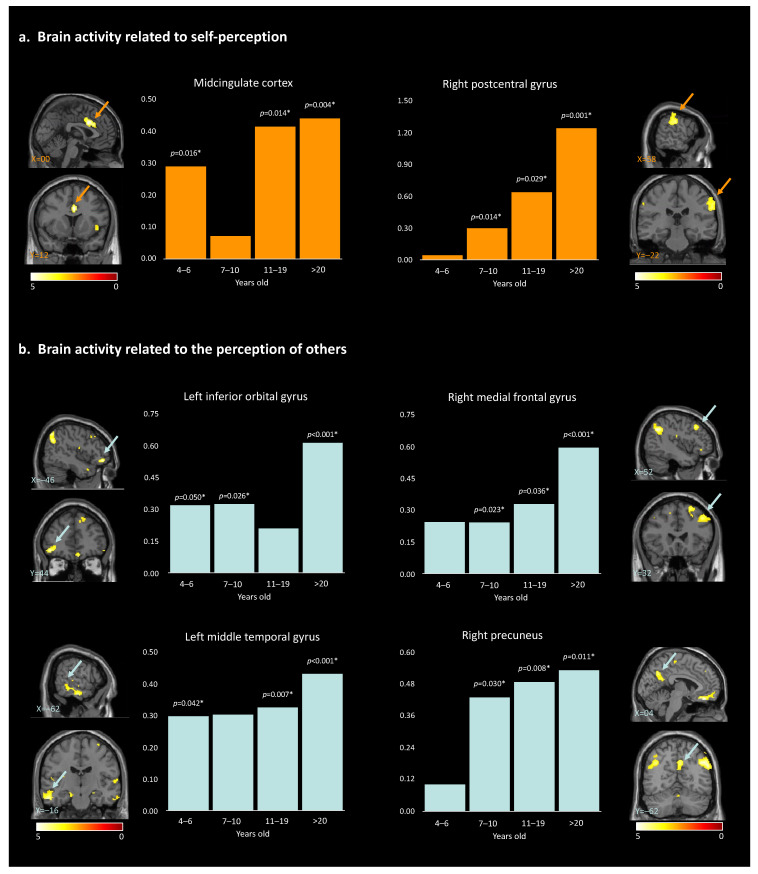
Results of the paired *t*-test analyses between the 4 different age groups, and the brain activity for self-perception (**a**) and for the perception of others (**b**). * indicate the significant *p*-values (>0.05). Arrows highlight the brain regions corresponding to the described area.

Hypothesis (3), developmental changes: For brain activity related to self-perception, the post hoc paired *t*-test to disentangle the age effect revealed that the MCC showed significant self-vs.-other perception discrimination in all age groups except the 7–10-year-olds. For the right postcentral gyrus, the self-vs.-other perception difference was observed in all age groups except the 4–6.5 y.o. group (Table 4, Figure 4a). Overall, self-vs.-other increased with age.

For brain activity related to the perception of ‘other’, post hoc paired *t*-tests revealed a global increase in discrimination between ‘other’ and self across development. This pattern was true for the right angular gyrus, left angular gyrus, right inferior temporal gyrus, right rectus, right precuneus, and right medial prefrontal cortex, starting at 7 years old. For the left middle temporal gyrus, neural activities significantly differed across all age groups except for the 7–10-year-old group. Within the right superior frontal gyrus, neural activities showed significant differences for other- than self-perception for the participants 11 years old and older. Finally, for the left inferior orbital gyrus, discrimination between others and self was observed in all age groups except for the 11–19-year-old group (Table 4, Figure 4b and Figure S1). The observed age-related differences in brain activity support the hypotheses of our study, suggesting that the neural mechanisms underlying self-perception and perception of others undergo developmental changes. While the global pattern is a linear increase in brain activity within specific regions, non-linear changes are observed for two age groups: 7–10-year-old and 11–19-year-old participants. These observations contribute to our understanding of social cognition and shed light on the complex relation between age, brain activation patterns, and the processing of self-vs.-other stimuli.

## 4. Discussion

Human beings are inherently social, and cooperation is essential for good life conditions. Consequently, we constantly read others’ actions, gestures, and faces, relying on the core capacity to discriminate self-vs.-other. With this study, we aimed to investigate age-related differences in brain activity during the processing of self and ‘other’ stimuli. Unveiling the emergence of self-vs.-other discrimination at the neural level across development could provide a rationale for how to adapt the social dimension of learning environments, for example. To this end, we compared brain activity during self-vs.-other stimuli, revealing a total of 11 brain regions whose activation maps significantly differ as a function of age groups.

Hypothesis (1), brain activity related to self-perception: First, two brain regions showed higher activation for the self than for the ‘other’ across development: the MCC and the right postcentral gyrus. These activation maps are consistent with past work, first showing the MCC to be involved in a wide range of social cognitive processes [47], including self-referential processing [48] and empathy [49] and second reporting the postcentral gyrus to be involved in the processing of sensory information from the body and playing a role in the formation of body image and self-awareness [50].

Hypothesis (2), brain activity related to ‘other’ perception: Our analysis revealed nine brain regions with significantly higher activation for perceiving others compared to oneself across development. These brain regions are all implicated in different fundamental aspects of social cognition, the mental processes used to make sense of the social world around us using multisensory skills: audiovisual speech or face–voice integrations (the angular gyrus and the left middle temporal gyrus; [51,52,53,54], somatosensory abilities (the MFG; [55], perception and processing of complex visual stimuli, including facial expressions and emotional states or body language (the ITG, [56,57]), self-processing [58], perspective-taking, empathy (the MFG), and the ToM (the precuneus; [29,59]). Additionally, social cognition relies on the ability to understand and respond to social cues, emotions, and the intentions of others (the right rectus; [1,60]) and implies working memory and spatial processing (the right superior frontal gyrus; [61,62,63,64]). Also, both the angular gyrus and the precuneus are considered key nodes of the default mode (DMN), a functional network of interconnected brain regions active during rest and self-referential mental activity [65] implicated in social cognition and ToM and more precisely in the ability to understand the mental states of others [66].

Hypothesis (3), developmental changes: The MCC showed higher activity for oneself than others across development, except in middle age children (i.e., 7–10 y.o.). The MCC is a key node in both the salience network and the social brain, two networks closely interconnected and working together to support social cognition and behavior [67]. The observed pattern could reflect a transient developmental shift, leading the MCC to be permeable to peers’ and self-related information similarly in 7–10 y.o. This could explain why spontaneous peer-to-peer interactions are observed at that age [68,69] and how these interactions could consequently improve their social skills. In line with this idea, a recent neuroimaging study revealed how 7–12 y.o. children activate specifically their salience network when watching peers but not adults performing unexpected actions [70]. Also, we observed that the activity within the right postcentral gyrus was increasingly higher for self-vs.-other stimuli from 7 years old on, highlighting a gradual gain in discriminative abilities across development. The postcentral gyrus is involved in the processing of sensory information from the body and may play a role in the formation of body image and self-awareness [50]. However, the lack of significant activation in the 4–6.5-year-old group suggests that the ability to distinguish between self and others at the sensorimotor level may not be fully developed at this age.

Except for the left inferior orbital gyrus, which showed a non-linear developmental pattern, increasing neural activities were observed in participants aged 7 years and older for the perception of others. Specifically, this higher activation was observed within the right and left angular gyrus, the right precuneus, the right inferior temporal gyrus (ITG), the right rectus, and the right medial frontal gyrus (MFG). Higher activation was also observed within the left middle temporal gyrus and the right superior frontal gyrus in participants beyond 10 years old. The findings of our study suggest a developmental trajectory of a gradual gain in discriminative abilities for others-versus-self. For example, it has been demonstrated that during childhood and adolescence there is a functional reconfiguration of the DMN, which coincides with the period of social and cognitive development [71]. There may be a link between the DMN and the representation of others in that they are both involved in processing self-referential information and constructing senses of self related to others (i.e., tailoring perceptions). Therefore, the increase in other-perception activation in these regions may reflect the growing ability to engage in perspective-taking, empathy, and ToM. Our results further highlighted a particularly significant increase in the level of MFG activation for the >20-year-old group when looking at others. One possible explanation is that older adults have greater social knowledge, which may enable them to process information about others in a more nuanced and sophisticated way. Therefore, these results could be linked to changes in the social environment or the types of social information that individuals are exposed to as they age. Alternatively, it could reflect changes in the way that older adults process social information, such as a greater focus on evaluating the emotional significance of social stimuli or a greater ability to integrate information from multiple sources.

Finally, the left inferior orbital gyrus showed a non-linear pattern, with a null difference between others- and self-perception in the 11–19 y.o. group. This brain region is known for being involved in reward value as well as emotion [45]. Emotional understanding is crucial for children’s successful social adjustment. In addition to developing appropriate social skills and prosocial responses to peers, children who are more successful at understanding emotions have a greater chance of forming positive interpersonal relationships that facilitate social adaptation [72,73]). As the processing of emotions changes across development, and during adolescence especially, it may become increasingly unstable and more intense [74].

Together, these results are consistent with previous research showing that self-referential processing is a fundamental aspect of human cognition and occurs early in development, continuing throughout life [17]. It happens concomitantly to the ability to discriminate oneself from others. However, we show that it is a complex and dynamic process. While this study provides strong results in terms of differences in activation maps, the sample size refrains from generalized claims. First, each age group had a limited sample size. As it is a clear challenge to have young children enrolled for an MRI study, it is even more complicated to address movement in a task-based fMRI experiment with 4- to 7-year-olds. However, future work should creatively find a way to overcome this aspect to replicate this work with a larger population, or at least use a longer task, as more diverse samples could offer a more comprehensive understanding of developmental patterns. Indeed, it is important to note that in our study, we specifically tested social partners of the same gender and ethnicity for the ‘other’ condition. However, this setup does not reflect real life where more diversity exists. To better mirror the complexities of social interactions, we could invent stimuli to test variations in age, gender, or ethnicity. This would allow us to explore how social enrichment influences the social brain at best across development. Additionally, the age groups were broadly defined (from 4-year-olds to adults) and divided into four categories. Exploring alternative ways to group the participants might reveal other developmental trends, considering the rapid cognitive changes that occur during childhood and adolescence. Also, we rely on fMRI as our primary imaging modality to capture neural responses; however, it could be interesting to complement our results with other neuroimaging techniques, such as electroencephalography (EEG), which could capture subtle reactions to social stimuli, providing a deeper comprehension of the dynamic temporal aspects of self-vs.-other processing. Finally, the study focuses on the brain activity captured during a specific fMRI task session, which might not fully capture the complexity of self-vs.-other processing in real life, considering its multifaceted nature. Future research could incorporate behavioral measures as well to complement the neuroimaging data.

To conclude, we showed here that while 4–6-year-old children have little discriminative skills, for both self and ‘other’ perceptions, a developmental shift seems to operate from 6 years on, with transient periods in specific neural activities up to adulthood. However, all share a fundamental capacity for introspection and self-reflection that is essential to our understanding of the human mind and behavior, even very young children. It is interesting to note that self-perception relies on two main brain regions, where perception of others seems to recruit and coordinate a broader range of brain regions. It may be more complex to identify and classify social cues than self-related cues.

These findings are important to better understand how social processing develops and consequently when, why, and which social context can support learning processes across childhood and teen years. Here, it seems that before 6 years of age, self-perception is the first pillar to develop. However, after 7 years of age, ‘other’ perception starts developing, suggesting a good age for introducing peer–peer learning. Finally, the teenage years seem to be a relevant developmental period for group learning.

## Figures and Tables

**Table 1:** Demographics.

Control Variable	4 to 6 y.o. (*n* = 9)	7 to 10 y.o. (*n* = 10)	11 to 19 y.o. (*n* = 10)	>20 y.o. (*n* = 10)	Overall (*n* = 39)
Age mean (SD)	5.30 (0.73)	7.52 (1.07)	15.85 (2.37)	24.67 (2.75)	13.54 (8.00)
Age min, max	4.10, 6.40	6.60, 9.70	11.90, 19.80	21.17, 31.00	4.10, 31.00
Gender ratio (f/m)	6/4	4/6	5/5	6/4	21/19

**Table 2:** Brain activity related to self-perception.

Localization	Coordinates (mm mm mm)			T	k_E_	q_FDR-corr_
Midcingulate cortex	0	12	32	5.28	273	0.001
Right postcentral gyrus	58	−22	32	4.60	414	0.001

**Table 3:** Brain activity related to ‘other’ perception.

Localization	Coordinates (mm mm mm)			T	k_E_	q_FDR-corr_
Right angular gyrus	42	−60	36	5.04	828	0.000
Left angular gyrus	−44	−68	48	4.96	504	0.000
Right inferior temporal gyrus	60	−10	−28	4.65	299	0.003
Left middle temporal gyrus	−62	−16	−24	4.77	452	0.000
Right rectus	4	38	−20	5.17	199	0.012
Right precuneus	4	−62	36	4.38	259	0.005
Right medial frontal gyrus	52	32	38	4.77	221	0.008
Right superior frontal gyrus	18	34	54	5.00	248	0.006
Left inferior orbital gyrus	−46	44	−10	5.11	136	0.046

**Table 4:** Developmental changes.

Brain Activity	4–6.5 y.o.	7–10 y.o.	11–19 y.o.	>20 y.o.
**Self-perception**				
Midcingulate cortex	T(8) = 2.58, *p* = 0.016	T(9) = 0.775, *p* = 0.229	T(9) = 2.63, *p* = 0.014	T(9) = 3.40, *p* = 0.004
Right postcentral gyrus	T(8) = 0.22, *p* = 0.416	T(9) = 2.60, *p* = 0.014	T(9) = 2.18, *p* = 0.029	T(9) = 4.18, *p* = 0.001
**Other-perception**				
Right angular gyrus	T(8) = −0.58, *p* = 0.288	T(9) = −1.86, *p* = 0.021	T(9) = −2.36, *p* = 0.021	T(9) = −4.57, *p* < 0.001
Left angular gyrus	T(8) = −1.02, *p* = 0.169	T(9) = −1.84, *p* = 0.050	T(9) = −3.07, *p* = 0.007	T(9) = −4.90, *p* < 0.001
Right inferior temporal gyrus	T(8) = −1.31, *p* = 0.113	T(9) = −2.30, *p* = 0.024	T(9) = −4.05, *p* = 0.001	T(9) = −4.90, *p* < 0.001
Left middle temporal gyrus	T(8) = −1.97, *p* = 0.042	T(9) = −1.77, *p* = 0.055	T(9) = −3.00, *p* = 0.007	T(9) = −4.59, *p* < 0.001
Right rectus	T(8) = −1.46, *p* = 0.091	T(9) = −2.21, *p* = 0.027	T(9) = −4.45, *p* < 0.001	T(9) = −3.34, *p* = 0.004
Right precuneus	T(8) = −0.54, *p* = 0.301	T(9) = −2.14, *p* = 0.030	T(9) = −2.99, *p* = 0.008	T(9) = −2.74, *p* = 0.011
Right medial prefrontal cortex	T(8) = −1.33, *p* = 0.110	T(9) = −2.32, *p* = 0.023	T(9) = −2.04, *p* = 0.036	T(9) = −4.34, *p* < 0.001
Right superior frontal gyrus	T(8) = −0.17, *p* = 0.433	T(9) = −1.66, *p* = 0.065	T(9) = −2.02, *p* = 0.037	T(9) = −7.91, *p* < 0.001
Left inferior orbital gyrus	T(8) = −1.86, *p* = 0.050	T(9) = −2.23, *p* = 0.026	T(9) = −1.50, *p* = 0.084	T(9) = −4.93, *p* < 0.001

## Data Availability

Data can be shared upon request to the corresponding author.

## Supplementary Materials

The following supporting information can be downloaded at: https://www.mdpi.com/article/10.3390/children10121914/s1, Table S1. MRI parameters. Figure S1. Results of the paired t-test analyses between the 4 different age groups, and the brain activity for the perception of others.

## References

[1] Ruby P., Decety J. (2004). How Would You Feel versus How Do You Think She Would Feel? A Neuroimaging Study of Perspective-Taking with Social Emotions. J. Cogn. Neurosci..

[2] Frith C.D. (2008). Social cognition. Philos. Trans. R. Soc. B Biol. Sci..

[3] Lang F.R., Reschke F.S. (2006). Social Relationships, Transitions, and Personality Development across the Life Span. Handbook of Personality Development.

[4] Young S.N. (2008). The neurobiology of human social behaviour: An important but neglected topic. J. Psychiatry Neurosci. JPN.

[5] De Mello C.B., Da Silva T., Cardoso G., Vinicius M., Alves C. (2023). Social Cognition Development and Socioaffective Dysfunction in Childhood and Adolescence. Social and Affective Neuroscience of Everyday Human Interaction: From Theory to Methodology.

[6] Yoon H.J., Seo E.H., Kim J.J., Han Choo I.L. (2019). Neural Correlates of Self-referential Processing and Their Clinical Implications in Social Anxiety Disorder. Clin. Psychopharmacol. Neurosci..

[7] Bahrick L.E. (2008). The Development of Perception in a Multimodal Environment. Theories of Infant Development.

[8] Falk A., Kosse F., Schildberg-Hörisch H., Zimmermann F. (2020). Self-Assessment: The Role of the Social Environment. SSRN Electron. J..

[9] Butterworth G. (1992). Origins of Self-Perception in Infancy. Source Psychol. Inq..

[10] Lewis M., Minar N.J. (2022). Self-Recognition and Emotional Knowledge. Eur. J. Dev. Psychol..

[11] Jacquey L., Fagard J., Esseily R., O’Regan J.K. (2020). Detection of sensorimotor contingencies in infants before the age of 1 year: A comprehensive review. Dev. Psychol..

[12] Rochat P. (1998). Self-perception and action in infancy. Exp. Brain Res..

[13] Amsterdam B. (1972). Mirror self-image reactions before age two. Dev. Psychobiol..

[14] Platek S.M., Thomson J.W., Gallup G.G. (2004). Cross-modal self-recognition: The role of visual, auditory, and olfactory primes. Conscious. Cogn..

[15] Lippé S., Kovacevic N., McIntosh A.R. (2009). Differential maturation of brain signal complexity in the human auditory and visual system. Front. Hum. Neurosci..

[16] Posner M.I., Rothbart M.K., Sheese B.E. (2007). The anterior cingulate gyrus and the mechanism of self-regulation. Cogn. Affect. Behav. Neurosci..

[17] Northoff G., Heinzel A., de Greck M., Bermpohl F., Dobrowolny H., Panksepp J. (2006). Self-referential processing in our brain—A meta-analysis of imaging studies on the self. NeuroImage.

[18] Steinbeis N. (2016). The role of self–other distinction in understanding others’ mental and emotional states: Neurocognitive mechanisms in children and adults. Philos. Trans. R. Soc. B Biol. Sci..

[19] Wellman H.M. (2014). Making Minds: How Theory of Mind Develops. Making Minds.

[20] Pfeifer J.H., Berkman E.T. (2018). The Development of Self and Identity in Adolescence: Neural Evidence and Implications for a Value-Based Choice Perspective on Motivated Behavior. Child Dev. Perspect..

[21] Rieffe C., Camodeca M. (2016). Empathy in adolescence: Relations with emotion awareness and social roles. Br. J. Dev. Psychol..

[22] Blakemore S.-J. (2008). The social brain in adolescence. Nat. Rev. Neurosci..

[23] Courtney A.L., Meyer M.L. (2020). Self-Other Representation in the Social Brain Reflects Social Connection. J. Neurosci..

[24] Giannouli V., Tsolaki M. (2023). Brain Volumes and Metacognitive Deficits in Knowledge of Self, Task and Strategies in Mathematics: A Preliminary Pilot One-Year Longitudinal Study in aMCI Patients Compared to Healthy Controls. Diagnostics.

[25] Devue C., Brédart S. (2011). The neural correlates of visual self-recognition. Conscious. Cogn..

[26] Molenberghs P., Johnson H., Henry J.D., Mattingley J.B. (2016). Understanding the minds of others: A neuroimaging meta-analysis. Neurosci. Biobehav. Rev..

[27] Ruby P., Decety J. (2003). What you believe versus what you think they believe: A neuroimaging study of conceptual perspective-taking. Eur. J. Neurosci..

[28] Saxe R.R., Whitfield-Gabrieli S., Scholz J., Pelphrey K.A. (2009). Brain regions for perceiving and reasoning about other people in school-aged children. Child Dev..

[29] Schurz M., Radua J., Aichhorn M., Richlan F., Perner J. (2014). Fractionating theory of mind: A meta-analysis of functional brain imaging studies. Neurosci. Biobehav. Rev..

[30] Moriguchi Y., Ohnishi T., Mori T., Matsuda H., Komaki G. (2007). Changes of brain activity in the neural substrates for theory of mind during childhood and adolescence. Psychiatry Clin. Neurosci..

[31] Carlson S.M., Koenig M.A., Harms M.B. (2013). Theory of mind. Wiley Interdiscip. Rev. Cogn. Sci..

[32] Beebe B., Lachmann F.M. (2003). The contribution of mother-infant mutual influence to the origins of self- and object representations. Psychoanal. Psychol..

[33] Brumariu L.E. (2015). Parent–Child Attachment and Emotion Regulation. New Dir. Child Adolesc. Dev..

[34] Araujo H.F., Kaplan J., Damasio A. (2013). Cortical midline structures and autobiographical-self processes: An activation-likelihood estimation meta-analysis. Front. Hum. Neurosci..

[35] Hu C., Di X., Eickhoff S.B., Zhang M., Peng K., Guo H., Sui J. (2016). Distinct and common aspects of physical and psychological self-representation in the brain: A meta-analysis of self-bias in facial and self-referential judgements. Neurosci. Biobehav. Rev..

[36] Murray R.J., Schaer M., Debbané M. (2012). Degrees of separation: A quantitative neuroimaging meta-analysis investigating self-specificity and shared neural activation between self- and other-reflection. Neurosci. Biobehav. Rev..

[37] Qin P., Northoff G. (2011). How is our self related to midline regions and the default-mode network?. NeuroImage.

[38] Tisserand A., Philippi N., Botzung A., Blanc F. (2023). Me, Myself and My Insula: An Oasis in the Forefront of Self-Consciousness. Biology.

[39] Asakage S., Nakano T. (2022). The salience network is activated during self-recognition from both first-person and third-person perspectives. Hum. Brain Mapp..

[40] Farrer C., Frith C.D. (2002). Experiencing Oneself vs Another Person as Being the Cause of an Action: The Neural Correlates of the Experience of Agency. NeuroImage.

[41] Jastorff J., Orban G.A. (2009). Human Functional Magnetic Resonance Imaging Reveals Separation and Integration of Shape and Motion Cues in Biological Motion Processing. J. Neurosci..

[42] Faghiri A., Stephen J.M., Wang Y.-P., Wilson T.W., Calhoun V.D. (2019). Brain Development Includes Linear and Multiple Nonlinear Trajectories: A Cross-Sectional Resting-State Functional Magnetic Resonance Imaging Study. Brain Connect..

[43] Conner I.P., Sharma S., Lemieux S.K., Mendola J.D. (2004). Retinotopic organization in children measured with fMRI. J. Vis..

[44] Peirce J.W. (2007). PsychoPy—Psychophysics Software in Python. J. Neurosci. Methods.

[45] Rolls E.T., Huang C.C., Lin C.P., Feng J., Joliot M. (2020). Automated anatomical labelling atlas 3. NeuroImage.

[46] Mazaika P.K., Hoeft F., Glover G.H., Reiss A.L. (2009). Methods and Software for fMRI Analysis for Clinical Subjects. AFNI Softw. Comp. Biomed. Res..

[47] Lou H.C., Luber B., Crupain M., Keenan J.P., Nowak M., Kjaer T.W., Sackeim H.A., Lisanby S.H. (2004). Parietal cortex and representation of the mental Self. Proc. Natl. Acad. Sci. USA.

[48] Denny B.T., Kober H., Wager T.D., Ochsner K.N. (2012). A Meta-Analysis of Functional Neuroimaging Studies of Self and Other Judgments Reveals a Spatial Gradient for Mentalizing in Medial Prefrontal Cortex. J. Cogn. Neurosci..

[49] Fan Y., Duncan N.W., de Greck M., Northoff G. (2011). Is there a core neural network in empathy? An fMRI based quantitative meta-analysis. Neurosci. Biobehav. Rev..

[50] Salvato G., Richter F., Sedeño L., Bottini G., Paulesu E. (2020). Building the bodily self-awareness: Evidence for the convergence between interoceptive and exteroceptive information in a multilevel kernel density analysis study. Hum. Brain Mapp..

[51] Bernstein L.E., Auer E.T., Wagner M., Ponton C.W. (2008). Spatio-temporal Dynamics of Audiovisual Speech Processing. NeuroImage.

[52] Joassin F., Pesenti M., Maurage P., Verreckt E., Bruyer R., Campanella S. (2011). Cross-modal interactions between human faces and voices involved in person recognition. Cortex.

[53] Seghier M.L. (2013). The angular gyrus: Multiple functions and multiple subdivisions. Neuroscientist.

[54] Pourtois G., de Gelder B., Bol A., Crommelinck M. (2005). Perception of Facial Expressions and Voices and of their Combination in the Human Brain. Cortex.

[55] Amodio D.M., Frith C.D. (2006). Meeting of minds: The medial frontal cortex and social cognition. Nat. Rev. Neurosci..

[56] Haxby Gobbini J.V., Furey M.I., Ishai M.L., Schouten A., Pietrini J.L. (2001). Distributed and overlapping representations of faces and objects in ventral temporal cortex. Science.

[57] Haxby J.V., Hoffman E.A., Gobbini M.I. (2000). The distributed human neural system for face perception. Trends Cogn. Sci..

[58] Cavanna A.E., Trimble M.R. (2006). The precuneus: A review of its functional anatomy and behavioural correlates. Brain.

[59] Schilbach L., Wohlschlaeger A.M., Kraemer N.C., Newen A., Shah N.J., Fink G.R., Vogeley K. (2006). Being with virtual others: Neural correlates of social interaction. Neuropsychologia.

[60] Hiser J., Koenigs M. (2018). The multifaceted role of ventromedial prefrontal cortex in emotion, decision-making, social cognition, and psychopathology. Biol. Psychiatry.

[61] du Boisgueheneuc F., Levy R., Volle E., Seassau M., Duffau H., Kinkingnehun S., Samson Y., Zhang S., Dubois B. (2006). Functions of the left superior frontal gyrus in humans: A lesion study. Brain.

[62] El-Baba R.M., Schury M.P. (2022). Neuroanatomy, Frontal Cortex.

[63] Job X., Kirsch L., Inard S., Arnold G., Auvray M. (2021). Spatial perspective taking is related to social intelligence and attachment style. Personal. Individ. Differ..

[64] Shelton A.L., Clements-Stephens A.M., Lam W.Y., Pak D.M., Murray A.J. (2011). Should social savvy equal good spatial skills? The interaction of social skills with spatial perspective taking. J. Exp. Psychol. Gen..

[65] Li W., Mai X., Liu C. (2014). The default mode network and social understanding of others: What do brain connectivity studies tell us. Front. Hum. Neurosci..

[66] Xie X., Mulej Bratec S., Schmid G., Meng C., Doll A., Wohlschläger A., Finke K., Förstl H., Zimmer C., Pekrun R. (2016). How do you make me feel better? Social cognitive emotion regulation and the default mode network. NeuroImage.

[67] Menon V., Uddin L.Q. (2010). Saliency, switching, attention and control: A network model of insula function. Brain Struct. Funct..

[68] Kington A., Gates P., Sammons P. (2013). Development of social relationships, interactions and behaviours in early education settings. J. Early Child. Res..

[69] Pellegrini A.D., Galda L., Flor D. (2011). Relationships, individual differences, and children’s use of literate language. Br. J. Educ. Psychol..

[70] Chiron A., Notter M., Eon Duval P., Décaillet M., Beaty R., Fornari E., Denervaud S. Observing unexpected actions from peers but not adults activates creativity-related brain processes.

[71] Fan F., Liao X., Lei T., Zhao T., Xia M., Men W., Wang Y., Hu M., Liu J., Qin S. (2021). Development of the default-mode network during childhood and adolescence: A longitudinal resting-state fMRI study. NeuroImage.

[72] Martins E.C., Osó A., Veríssimo M., Martins C. (2016). Emotion understanding in preschool children: The role of executive functions. Int. J. Behav. Dev..

[73] Rosnay M., de Harris P.L., Pons F. (2013). Emotion understanding and developmental psychopathology in young children. Social Cognition and Developmental Psychopathology.

[74] Bailen N.H., Green L.M., Thompson R.J. (2018). Understanding Emotion in Adolescents: A Review of Emotional Frequency, Intensity, Instability, and Clarity. Emot. Rev..

