# Supplementary material for: Me, Myself, and I: Neural Activity for Self versus Other across Development
Source: Children (Basel). 2023 Dec 12;10(12):1914. doi: 10.3390/children10121914 (PMC10742061; doi:10.3390/children10121914)

Supplement information

Table S1. MRI parameters

	MP-RAGE	EPI_fMRI
**Number of Slices**	208	64
**Slice thickness (mm)**	1	2
**Distance factor (%)**	0	0
**FOV Read (mm)**	256	200
**FOV Phase (mm)**	256	200
**Repetition Time (ms)**	2000	1000
**Echo Time (ms)**	2,47	30
**Inversion Time (ms)**	900	x
**Flip Angle (deg)**	8	80
**Fat Suppression**	x	Yes
**Number of measurements**	x	180
**Base Resolution**	256	100
**Phase Resolution**	256	100
**Phase Partial Fourier**	7\8	Off
**Slice Partial Fourier**	7\8	Off
**Interpolation**	Off	Off
**Acceleration mode**	Grappa	SimultaneousMultiSlices (SMS)
**Acceleration factor**	2	2
**Acceleration factor Slice**	x	4
**Acceleration Refrence Lines**	24	24
**B0 Shim mode**	Tune Up	Standard
**B1 Shim mode**	TrueForm	TrueForm
**Echo spacing (ms)**	7,4	0,57
**Bandwidth (Hz/Px)**	200	2272
**EPI factor**	x	100
**Gradient Mode**	Whisper	Performance
**Voxel Size (mm)**	1x1x1	2x2x2
**Acquisition Time**	4min06sec	3min12sec

Figure S1. Results of the paired t-test analyses between the 4 different age groups, and the brain activity for the perception of others.

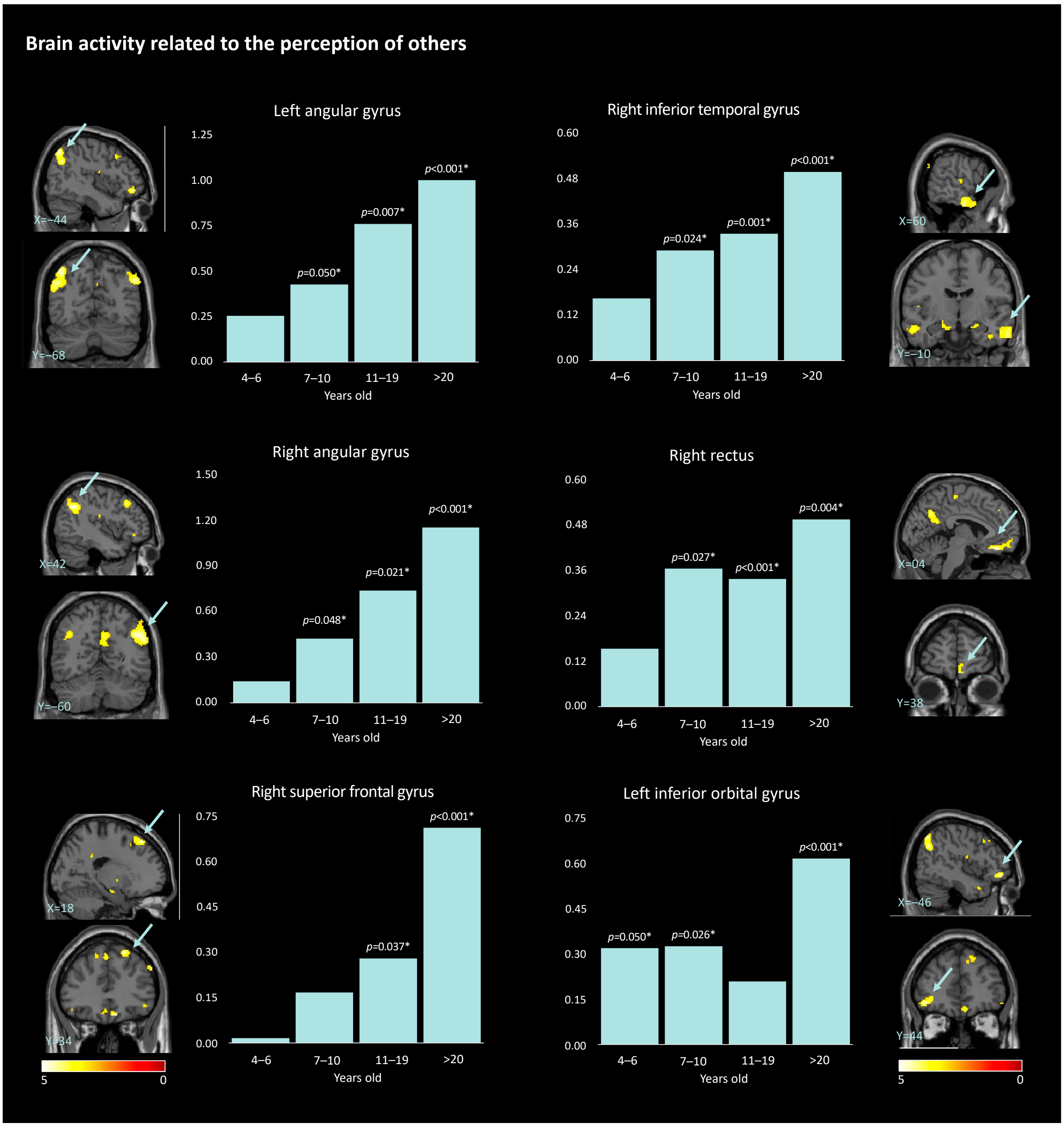

Supplement: Supplementary file 1 [file children-10-01914-s001.zip › children-2699015-supplementary.pdf]
